# Phylogeography of a widespread small carnivore, the western spotted skunk (*Spilogale gracilis*) reveals temporally variable signatures of isolation across western North America

**DOI:** 10.1002/ece3.2931

**Published:** 2017-05-03

**Authors:** Adam W. Ferguson, Molly M. McDonough, Gema I. Guerra, Margaret Rheude, Jerry W. Dragoo, Loren K. Ammerman, Robert C. Dowler

**Affiliations:** ^1^ Department of BiologyTexas Tech UniversityLubbockTXUSA; ^2^ Division of MammalsNational Museum of Natural HistorySmithsonian InstitutionWashingtonDCUSA; ^3^ Center for Conservation GenomicsSmithsonian Conservation Biology InstituteNational Zoological ParkWashingtonDCUSA; ^4^ Department of BiologyAngelo State UniversitySan AngeloTXUSA; ^5^ United States Fish and Wildlife ServiceTwin Cities Ecological Services OfficeBloomingtonMNUSA; ^6^ Museum of Southwestern BiologyUniversity of New MexicoAlbuquerqueNMUSA

**Keywords:** Carnivora, desert southwest, ecological niche modeling, Mephitidae, mitochondrial DNA, pre‐Pleistocene divergence, Quaternary climate change, refugia

## Abstract

We analyzed phylogeographic patterns in the western spotted skunk, *Spilogale gracilis* Merriam, 1890 (Carnivora: Mephitidae) in relation to historical events associated with Pre‐Pleistocene Divergence (PPD) and Quaternary climate change (QCC) using mitochondrial DNA from 97 individuals distributed across Western North America. Divergence times were generated using BEAST to estimate when isolation in putative refugia occurred. Patterns and timing of demographic expansion was performed using Bayesian skyline plot. Putative climatic refugia resulting from Quaternary climate change were identified using paleoecological niche modeling and divergence dates compared to major vicariant events associated with Pre‐Pleistocene conditions. We recovered three major mitochondrial clades corresponding to western North America (California, Baja, and across the Great Basin), east‐central North America (Texas, central Mexico, New Mexico), and southwestern Arizona/northwestern Mexico. Time to most recent common ancestor for *S. gracilis* occurred ~1.36 Ma. Divergence times for each major clade occurred between 0.25 and 0.12 Ma, with signature of population expansion occurring 0.15 and 0.10 Ma. Ecological niche models identified three potential climatic refugia during the Last Interglacial, (1) west coast of California and Oregon, (2) northwestern Mexico, and (3) southern Texas/northeastern Mexico as well as two refugia during the Last Glacial Maximum, (1) western USA and (2) southern Texas/northeastern Mexico. This study supports PPD in shaping species‐level diversity compared to QCC‐driven changes at the intraspecific level for *Spilogale*, similar to the patterns reported for other small mammals (e.g., rodents and bats). Phylogeographic patterns also appear to have been shaped by both habitat and river vicariance, especially across the desert southwest. Further, continuing climate change during the Holocene coupled with anthropogenic modifications during the Anthropocene appears to be removing both of these barriers to current dispersal of western spotted skunks.

## 1 Introduction

Recent phylogeographic studies of a diverse array of North American plants and animals have resulted in identification of broadscale patterns of genetic variation attributable to both Pre‐Pleistocene Divergence (PPD) resulting from vicariance and Quaternary climate change (QCC) associated with expansion and contraction of glaciers during the Pleistocene‐Holocene (Hewitt, 2004). Although the relative roles of each of these forces in shaping patterns of speciation have been debated, with some arguing QCC has little if anything to do with species‐level evolution (Bennett, 2004) while others support lineage‐specific impacts of QCC on vertebrate speciation (Peterson & Ammann, 2013), newer methodologies including ecological niche modeling (ENM) and molecular dating of divergence times has revolutionized our ability to characterize species‐specific responses to both PPD and QCC (Alvarado‐Serrano & Knowles, 2014; Hickerson et al., 2010). For western North America, a region of intense phylogeographic study (Swenson & Howard, 2005), PPD tends to be characterized by vicariant, tectonic events of the Neogene such as mountain orogensis or sea level inundation, although species currently inhabiting the region display mixed evolutionary responses to such barriers (Graham, Hendrixson, Hamilton, & Bond, 2015). In contrast, the impact of QCC on species evolution is characterized by refugia creation via range fragmentation followed by subsequent expansion in response to changing climates, often resulting in regionally defined responses by animals adapted to particular habitats (e.g., rodents of the warm deserts, Riddle & Hafner, 2006). Additional lines of evidence continue to refine locations of such Pleistocene refugia, enhancing our ability to interpret phylogeographic patterns for well‐studied regions (Holmgren et al., 2014). Thus, species‐level studies continue to add value to investigations of shared phylogeographic patterns related to PPD and QCC across broad geographic areas, even for taxa with previously published phylogeographic studies (Bryson, Jaeger, Lemos‐Espinal, & Lazcano, 2012; Jaeger, Riddle, & Bradford, 2005).

Phylogeographic patterns and processes across western North America have been well‐characterized for three regions in particular, (1) northwestern North America (as defined by Shafer, Cullingham, Cote, & Coltman, 2010), (2) the warm deserts (i.e., Chihuahuan, Mojave, and Sonoran Deserts as defined by Riddle & Hafner, 2006), and (3) the Great Basin Desert (as defined by Riddle, Jezkova, Hornsby, & Matocq, 2014). For northwestern North America, animals were originally thought to have been isolated between two major refugia during glacial maximum, Berengia and the Pacific Northwest (Pielou, 1991). However, a more complex phylogeographic pattern has been proposed, indicating, for example, that both PPD events such as the orogeny of the Cascade‐Sierra mountain chain 5‐2 Ma and QCC‐driven refugia have played a role in shaping genetic patterns of the regions flora and fauna (Shafer et al., 2010). Similarly for North America's warm deserts, both PPD and QCC may have contributed to patterns of genetic subdivision, with PPD‐associated late Miocene‐Pliocene vicariant events such as the formation of the Bouse Embayment potentially leading to speciation via allopatry followed by subsequent intraspecies‐level divergence attributable to QCC events such as the climatically driven expansion and contraction of the Deming Plains (Hafner & Riddle, 2011; Riddle & Hafner, 2006). Finally, mammals and other biota in the Great Basin Desert have phylogeographic structure that appears to be first shaped by PPD and then followed by dramatic alterations to species’ ranges attributable to QCC (Riddle et al., 2014).

Although a multitude of studies on animal phylogeography exists for western North America, most have focused on only one of three particular regions—northwestern North America, warm deserts, and Great Basin—with few recent studies addressing how different biogeographic histories associated with each region function in concert to shape patterns of variation within a single species (Harding & Dragoo, 2012; Latch, Reding, Heffelfinger, Alcalá‐Galván, & Rhodes, 2014; Nava‐García, Guerrero‐Enríquez, & Arellano, 2016). However, recent work on wide‐ranging species whose distributions encompass all or parts of these three regions have begun to emerge, including work on both large [e.g., mule deer *Odocoileus hemionus* (Latch et al., 2014), bighorn sheep *Ovis canadensis* (Buchalski et al., 2016); black bear *Ursus americanus* (Puckett, Etter, Johnson, & Eggert, 2015)] and small [e.g., Antelope squirrels of the genus *Ammospermophilus* (Mantooth, Hafner, Bryson, & Riddle, 2013); harvest mouse *Reithrodontomys megalotis* (Nava‐García et al., 2016)] mammals. These shed light on how wide‐ranging, vertebrate taxa with broad ecological niches respond to biogeographic events associated with different historical signatures. Not surprisingly, larger, more mobile species tend to maintain weaker levels of genetic subdivisions (Latch et al., 2014) than smaller species (Mantooth et al., 2013) but see (Nava‐García et al., 2016) across these regions. Patterns associated with mid‐sized mammals remain obscure for much of this region, including important yet often neglected members of these ecological communities, such as small carnivores (Prugh et al., 2009; Roemer, Gompper, & Van Valkenburgh, 2009).

One notable group of carnivores that remains understudied, especially in western North America, despite their diverse ecologies and broad distributions across important biogeographical areas is the skunks (Carnivora, Mephitidae, Dragoo, 2009). One genus of skunks, *Spilogale*, is represented by four extant taxa (Dragoo, 2009): the western spotted skunk (*S. gracilis*), eastern spotted skunk (*S. putorius*), southern spotted skunk (*S. angustifrons*), and pygmy spotted skunk (*S. pygmaea*). Members of *Spilogale* have existed in North America since the early Pliocene (Van Gelder, 1959), with the oldest fossils of the extinct Rexroad spotted skunks, *S. rexroadi* from Kansas and Texas dating back to *c*. 3.0–3.5 Ma (Dalquest, 1971; Hibbard, 1941a,b). The western spotted skunk, *S. gracilis*, is a small (200–800 g) species currently distributed from central Mexico north to British Columbia, with an east–west distribution reaching from the California coast to the central Great Plains (Verts, Carraway, & Kinlaw, 2001).

**Figure 1:**
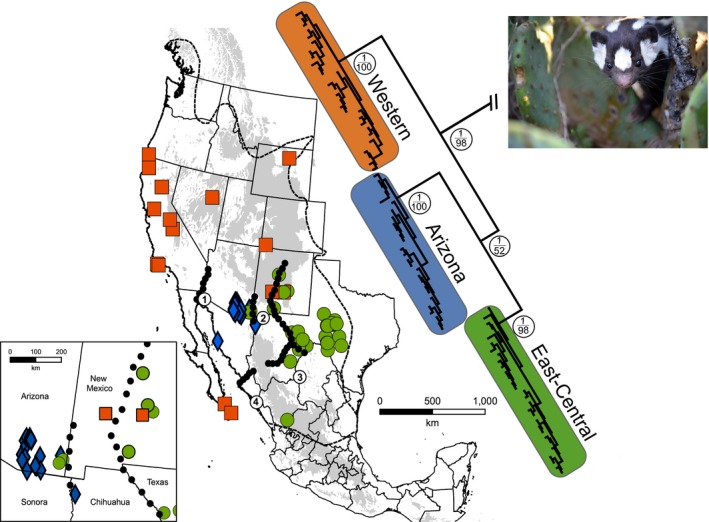
Phylogeographic patterns estimated using Bayesian inference for *Spilogale gracilis* based on Cytb mitochondrial gene. Nodal support for the major clades depicted in the open circle (Bayesian posterior probability above and maximum‐likelihood bootstrap below). Shading indicates regions with elevations >1,700 m. Numbers refer to historical barriers associated with phylogeographic subdivisions in warm desert taxa: (1) Boues Embayment/Colorado River, (2) Deming Plains, (3) Rio Grande‐Rio Conchos, and (4) Sierra Barabampo‐Rio Fuerte adapted from Riddle and Hafner (2006). Map of clades projected in Mollweide with a WGS84 datum. Colored clades correspond to respective mitochondrial DNA clades referred to in text as Arizona (Blue, diamonds), East‐Central (Green, circles) and West (Orange, squares). Photograph of a western spotted skunk from San Angelo, Texas. Photograph Credit: Robert C. Dowler

The broad geographic distribution of *S. gracilis* across three well‐characterized regions of western North America (Figure 1) indicates that the evolutionary history of this species could have been shaped by a number of PPD events, including but not limited to mountain orogeny (e.g., Rocky Mountains, Sierra Nevada, and Sierra Madre Occidental), river drainage alterations (e.g., Rio Grande, Rio Conchos, and Rio Nazas), and sea level changes (e.g., inundation along the Colorado River forming the Bouse Embayment). Characterization of these PPD events for each respective region and their potential impacts on phylogeographic patterns in a wide array of plants and animals provides useful hypotheses for testing within *S. gracilis*. In addition, habitats across the range of *S. gracilis* are characterized by changes in extent and distribution associated with fluctuating climates of the Quaternary. Thus, the catholic choice of habitat maintained by *S. gracilis* also makes it well‐suited for examining patterns of genetic subdivision associated with QCC. This broad ecological niche combined with the estimated age and distribution of this lineage across a well‐studied but complex biogeographic region makes this species well‐suited for analyses of the relative influence of PPD and QCC in shaping phylogeographic patterns of small carnivores across western North America.

Using mitochondrial DNA (mtDNA) and ecological niche modeling, we explored patterns of genetic variation and divergence time estimates for *S. gracilis* and other *Spilogale* species in relation to well‐characterized events associated with PPD as well as fluctuating climates attributable to QCC across western North America. We predict that *S. gracilis* will show phylogeographic patterns intermediate to those of larger, more mobile species and smaller, less mobile species, with species‐level divergence occurring around well‐defined phylogeographic breaks associated with PPD followed by population‐level divergence associated with QCC.

## 2 Materials and Methods

### 2.1 Sampling and laboratory methods

Genetic data from recent field expeditions and museum tissue collections were obtained from 97 individual *S. gracilis* representing 78 unique localities (see Appendix S1, Table S1.1). All genetic sequences were deposited onto GenBank together with associated voucher specimen information, when available (KY679911–KY680207). Sampling represented 6 of 7 recognized subspecies of *S. gracilis* including *S. g. amphialus*,* S. g. gracilis*,* S. g. latifrons*,* S. g. leucoparia*,* S. g. lucasana*, and *S. g. phenax* (Dragoo, 2009). Our samples covered the entire distribution of *S. gracilis* with dense sampling across the boundary of the Sonoran/Chihuahuan Deserts. For genetic analysis, we amplified and sequenced three mtDNA fragments: the complete cytochrome‐*b* (Cytb; 1140 bps), partial NADH dehydrogenase 5 (ND5; 629 bps), and partial control region (D‐Loop; 503 bps; see Appendix S2 in Supporting Information for protocols and Table S2.1 for primer sequences). DNA sequence alignments were estimated using sequencher 4.9 (Gene Codes Corporation, Ann Arbor, MI, USA). Cycle sequencing was performed using bigdye version 3.1 (Applied Biosystems, Foster City, CA, USA) following the manufacturer's protocols. Sequences were electrophoresed on an ABI‐3100 or 3130 (Applied Biosystems). Models of evolution for each gene were selected using mega 6 (Tamura, Stecher, Peterson, Filipski, & Kumar, 2013) based on BIC and AICc scores.

### 2.2 Phylogenetic estimates

Phylogenetic trees were estimated using Bayesian Inference with mrbayes 3.2.2 (Ronquist et al., 2012) within the CIPRES Science Gateway (Miller, Pfeiffer, & Schwartz, 2010). Two MCMC chains were run for 10 million generations, sampling trees every 100 iterations and using a burn‐in period of 10,000 trees. Base frequencies were assigned an equal Dirichlet distribution (1.0, 1.0, 1.0, 1.0). A maximum‐likelihood phylogeny was estimated using phyml version 3.0 (Guindon et al., 2010). The BIONJ option was selected for the starting tree (Gascuel, 1997), and 1,000 bootstrap replicates were performed to estimate nodal support. Proportion of invariable sites and the shape of the gamma distribution were estimated within the maximum‐likelihood framework. Phylogenetic trees were estimated for individual mitochondrial genes for Cytb and D‐loop only and a concatenated dataset of Cytb and D‐loop combined.

### 2.3 Divergence time estimates

Divergence times were estimated using the three‐gene dataset (Cytb, ND5, and D‐Loop) in beast version 2.0 (Drummond, Suchard, Xie, & Rambaut, 2012). A likelihood ratio test for a molecular clock (Felsenstein, 1988) performed in mega6 was rejected, and therefore, an uncorrelated log‐normal relaxed clock (representing heterogeneous evolutionary rates) was employed. Both birth‐death (Gernhard, 2008) and Yule (Yule, 1925) speciation priors were independently evaluated using Bayes factor tests following Suchard, Weiss, and Sinsheimer (2001). Substitution models were unlinked and models of evolution selected in the phylogenetic analysis were used as priors. Taxon sampling included three individuals from each major *S. gracilis* clade recovered from the phylogenetic analysis as well as the following outgroup taxa: *Mydaus javanensis/marchei, Conepatus leuconotus, Mephitis mephitis, Spilogale putorius,* and *Spilogale pygmaea* (See Appendix S1, Table S1.1).

Divergence time estimates were based on three priors, one fossil, and two molecular. The fossil prior was used to inform time to most recent common ancestor (TMRCA) for all *Spilogale* (offset of 3 Ma), based on the oldest *Spilogale* fossil (Hibbard, 1941a,b; Dalquest, 1971) with an exponential distribution with a mean of 1.63 such that 97.5% of the prior probability was below 9 Ma, a date which corresponds to the origin of Mephitidae in North America (Wang, Carranza‐Castañeda, & Aranda‐Gómez, 2014; Wang, Whistler, & Takeuchi, 2005). The second prior was based on a molecular estimate of TMRCA for the three extant genera of New World mephitids (*Conepatus*,* Mephitis*, and *Spilogale*) and was set using a normal distribution (mean = 9.2, *SD* = 1.7) to place the 2.5 and 97.5 quantiles between 6 and 13 Ma, the highest posterior density (HPD) interval associated with this molecular date (Eizirik et al., 2010). Finally, tree height was given a prior of 20 Ma based on TMRCA between *Mydaus* and extant New World skunks (Eizirik et al., 2010).

### 2.4 Demographic analysis

Demographic patterns were estimated using a Bayesian skyline plot (BSP; (Heled & Drummond, 2008) in beast 1.8 (Drummond et al., 2012). For *S. gracilis,* a BSP was estimated using the three‐gene dataset for 13 individuals. Mutation rates estimated from previous BEAST analysis were used to inform prior distribution in BSP using a log‐normal relaxed clock with a normally distributed tree height prior corresponding to MRCA for all *S. gracilis* (mean = 0.98, SD = 0.2). The analysis consisted of 10 million iterations sampled every 1,000 iterations. For all BEAST analyses, effective sample sizes of posterior parameters were evaluated using tracer 1.5 (Rambaut, Suchard, & Drummond, 2009). Final figures were made in tracer (for BSP) and figtree 1.4.2 (Rambaut, 2015).

### 2.5 Ecological niche modeling

#### 2.5.1 Present‐day models

Ecological niche models (ENM) were generated following the Biotic, Abiotic, and Movement (BAM) conceptual framework (Soberón & Peterson, 2005). Estimation of abiotically suitable conditions under this framework implies delimitation of the Grinnellian or scenopoetic fundamental niche (Soberón, 2007) and requires explicit assumptions regarding model input and interpretation (Soberón & Peterson, 2005), thereby providing a more transparent and repeatable methodological approach to ENM development (McDonough et al., 2015).

For *S. gracilis*, we initially defined “M” or the region of the world accessible to this species since its origin (Soberón & Peterson, 2005), as a 500‐km buffer around all localities (See Appendix S3, Fig. S3.1). Fossil records of *S. gracilis* from Alberta, Canada (Kurten & Anderson, 1980), were used to demarcate the northeastern extent of the training area while the eastern edge was limited to the western most boundaries of the congener, *S. putorius*. The southern edge was truncated along the northern border of the distribution of the southern spotted skunk (*S. angustifrons*). All range boundaries were interpreted from expert generated distribution maps downloaded from the IUCN Redlist (www.iucnredlist.org/).

Occurrence records were obtained from museum voucher specimens via the Mammal Networked Information System (MaNIS; http://manisnet.org/) or from tag information collected from visited collections or provided by curators and collection managers (see Acknowledgments and Appendix S3, Table S3.1). All textual locality information was georeferenced following best‐practices guidelines (Chapman & Wieczorek, 2006) and only specimens with a coordinate uncertainty less than 5 km were used in model development. To reduce potential biases associated with clustered sampling localities, occurrence data were spatially thinned by selecting a random subset of >100 occurrence records with a minimum distance between records of 0.6111 decimal degrees (~50 km) in ArcGIS 10.1 (ESRI, Redland, CA). This reduction in data was performed three times, and each subset was used to generate its own unique ENM. However, due to the disproportionate number of specimens from the 255 unique occurrence records found across the distribution of *S. gracilis*, spatially thinned subsets resulted in models that tended to over‐predict suitability in regions associated with more records (e.g., California, Texas, see Appendix S3, Fig. S3.1). Thus, a manual thinning process whereby we selected a subset of records evenly distributed across the range of *S. gracilis* was used to generate final ENMs. This process yielded 84 unique occurrence records that were used to generate final models of the abiotic niche of *S. gracilis* (see Appendix S3, Table S3.1).

Present (1950–2010) environmental data in the form of bioclimatic variables were accessed from WorldClim (www.worldclim.org; Hijmans, Cameron, Parra, Jones, & Jarvis, 2005) at a spatial resolution of 2.5′ (~5 × 5 km). This coarser resolution was chosen over the available 30 arc‐second data to better match the 5‐km uncertainties associated with georeferenced specimen‐locality coordinates. Although utilizing all 19 variables without addressing issues associated with correlated variables has been identified as a concern in ENM (Guisan & Thuiller, 2005), we chose to include all 19 variables in model development following reasons outlined in McDonough et al. (2015).

Models were generated using the maximum entropy algorithm in maxent 3.3.3 (Phillips, Anderson, & Schapire, 2006). Maxent models were generated under the “autofeatures”’ option, allowing for linear, quadratic, product, threshold, and hinge features to be used to describe relationships between locations and environmental conditions (Merow, Smith, & Silander, 2013) with both clamping and extrapolating options deselected (Owens et al., 2013). Although raw outputs were recently suggested as preferable to logistic output within maxent, given assumptions associated with τ, logistic output was chosen to allow for comparisons across species with inherently different model calibration regions (Ferguson, 2014; Merow et al., 2013). A total of 10 bootstrap model replicates were used to generate predictions of suitable conditions with final models representing an average of these 10 replicates. All raster processing and assessments were performed using a combination of arcGIS 10.1 and the R statistical software environment (http://www.r-project.org/).

#### 2.5.2 Historic models

The resulting predictive models developed for present‐day conditions were used to project suitable environmental conditions onto two Pleistocene climatic coverages, representing the last glacial maximum (LGM; ~21,000 years BP) and the last interglacial (LIG; ~120,000–140,000 years BP) periods, respectively. Environmental layers for both LGM and LIG were accessed from the WorldClim database (http://www.worldclim.org/past). LGM coverages are provided at 2.5 arc‐minutes resolution for two climate models: the Community Climate System Model (CCSM, http://www.ccsm.ucar.edu/; (Kiehl & Gent, 2004) and the Model for Interdisciplinary Research on Climate (miroc, ver. 3.2 http://www.ccsr.u-tokyo.ac.jp/~hasumi/MIROC/). These general circulation models are derived from the Palaeoclimate Modelling Intercomparison Project Phase II (PMIP2; http://pmip2.lsce.ipsl.fr/) database. LIG coverages originating from Otto‐Bliesner et al. (2006) are provided at 30 arc‐seconds and thus were rescaled to 2.5 arc‐minutes in arcGIS 10.1 to match the present and LGM resolutions of 2.5 arc‐minutes (~5 km). Results generated for both CCSM and MIROC circulation models were qualitatively similar (data not shown); thus, only CCSM was used to develop subsequent models.

## 3 Results

### 3.1 Mitochondrial phylogeny and geographic distribution of genetic variation

Phylogenetic analyses of the Cytb dataset (*n* = 97) recovered three well‐supported clades within *S. gracilis*, subsequently referred to as West, East‐Central, and Arizona (Figure 1). These clades differed from one another with interclade Kimura‐2 parameter (K2P) genetic distances ranging from 0.032 to 0.043, compared to 0.074–0.189 for outgroup taxa (Table 1). Individual gene trees for Cytb and D‐loop recovered the three clades as did the concatenated 2‐gene (Cytb + D‐loop) dataset. ND5 was not included in phylogenetic estimates due to low sample size and minimal geographic coverage (see Appendix S1, Table S1.1). Given the depth of sampling for the Cytb tree and the congruency seen among D‐loop and the concatenated dataset, we chose to present only the Cytb phylogeny.

**Table 1:** Average Kimura‐2 parameter genetic distances within (diagonal) and between mitochondrial clades of *Spilogale gracilis* for the Cytb gene only, estimated using mega 6.0

Clade	East‐Central	West	Arizona	*Spilogale putorius*	*Spilogale pygmaea*	*Conepatus leuconotus*	*Mephitis mephitis*
East‐Central	0.004						
West	0.043	0.002					
Arizona	0.032	0.043	0.003				
*S. putorius*	0.074	0.083	0.084	–			
*S. pygmaea*	0.163	0.160	0.163	0.151	–		
*C. leuconotus*	0.189	0.183	0.187	0.185	0.171	–	
*M. mephitis*	0.142	0.152	0.149	0.141	0.147	0.177	–

Geographically, the West clade (*n* = 32) was distributed from Baja del Sur north through California (including the Channel Islands) and into southern Oregon, with additional individuals from Colorado, Nevada, Wyoming, and a single individual from western New Mexico (Figure 1). The East‐Central clade (*n* = 37) included samples ranging from central Mexico (Durango) north through west‐central Texas and, in New Mexico, as far east as the Rio Grande River. However, two individuals from the Chiricahua Mountains in Cochise County Arizona were the only samples from this clade found west of the Rio Grande and the Deming Plains, both notable Pleistocene barriers for this region (Hafner & Riddle, 2011; Riddle & Hafner, 2006). The Arizona clade (*n* = 27) was restricted to southeastern Arizona, the extreme northwestern corner of Chihuahua, and the west‐central coast of Sonora.

### 3.2 Divergence times of major mtDNA lineages

**Figure 2:**
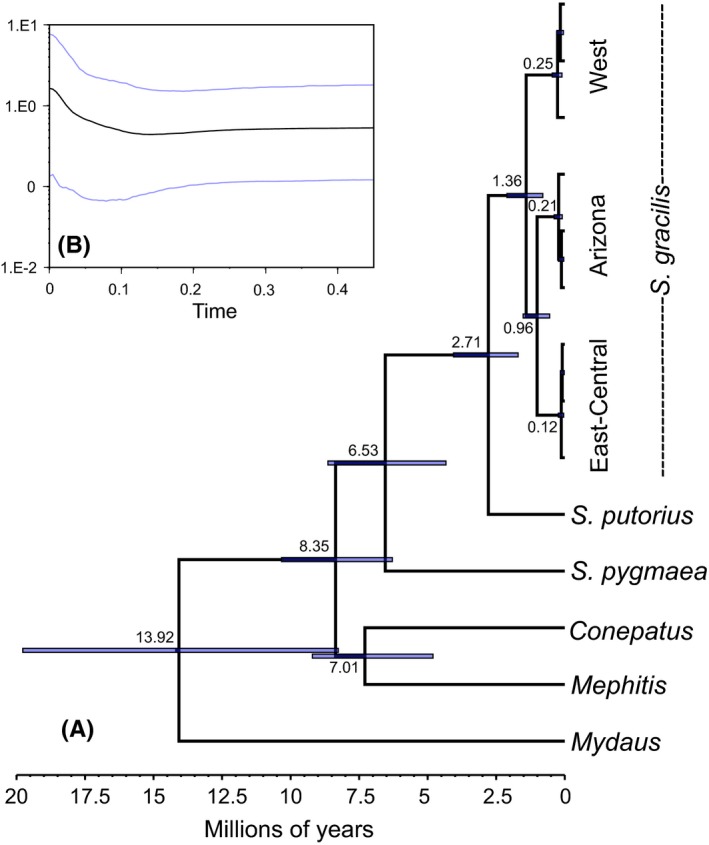
Timescale of diversification for Mephitidae based on mitochondrial DNA coalescent modeling estimated in BEAST using the three‐gene concatenated dataset (Cytb, ND5, and D‐loop). Ultrametric tree with median ages and 95% HPDs depicted at the nodes (a). Bayesian skyline plot for *Spilogale gracilis* showing signature of population expansion through time (b)

TMRCA for all *Spilogale* from the three‐gene dataset (Cytb, ND5, and D‐loop) was estimated at *c*. 6.53 Ma (95% HPD = 8.64–4.34 Ma; Table 2, Figure 2). TMRCA for *S. gracilis* and *S. putorius* occurred *c*. 2.71 Ma (4.06–1.70), indicating that these species diverged from one another during the late Pliocene to early Pleistocene. These estimates indicate that a majority of diversification at the interspecific level within the genus *Spilogale* occurred as a result of events associated with PPD. TMRCA for all *S. gracilis* clades was *c*. 1.36 Ma (95% HPD = 2.11–0 .82; Table 2, Figure 2). This estimate, with an HPD confined entirely to the Pleistocene, indicates that intraspecific diversification within *S. gracilis* appears to be attributable to isolation resulting from QCC and not PPD. Coalescent modeling revealed strong support for the sister relationship between East‐Central and Arizona clades, estimated to share a common ancestor approximately 0.96 Ma (95% HPD = 0.55–1.52 Ma). Divergence times for West, Arizona, and East‐Central clades ranged from *c*. 0.25 to 0.12 Ma.

**Table 2:** The time to most recent common ancestor (TMRCA) and 95% highest posterior density (HPD) estimates for selected mitochondrial clades of *Spilogale gracilis* (Clade Identifier) and included outgroups based on the three‐gene dataset (Cytb, ND5, D‐loop)

MRCA	Ma	95% HPD
All *Spilogale gracilis* (East* *+ West* *+ Arizona)	1.36	2.11–0.80
TX, NM, Chihuahua* *+ AZ, Sonora (East* *+ Arizona)	0.96	1.52–0.55
TX, NM, Chihuahua (East)	0.12	0.23–0.04
CA, CO, NM, NV, OR, WY, Baja del Sur (West)	0.25	0.46–0.10
AZ, Sonora (Arizona)	0.21	0.39–0.09
*S. gracilis *+ *S. putorius*	2.71	1.35–3.82
*S. gracilis *+ *S. putorius *+ *S. pygmaea*	6.53	8.64–4.34

### 3.3 Historical demography

Demographic reconstruction using BSP indicated a population increase for all clades starting between *c*. 0.15 and 0.10 Ma with continued population increase into the present (Figure 2).

### 3.4 Ecological niche modeling

#### 3.4.1 Present‐day models

**Figure 3:**
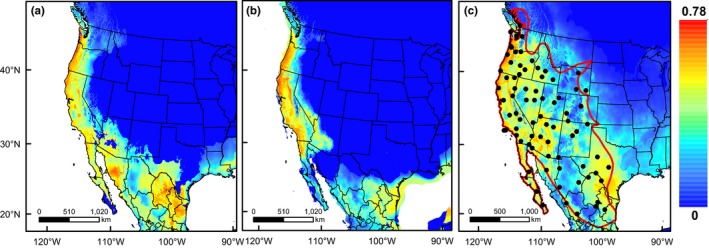
Depictions of ecological niche models generated using the maxent software package for *Spilogale gracilis* for (a) Last Interglacial (~0.12 Ma), (b) Last Glacial Maximum (~0.02 Ma), and (c) present‐day (1950–2000) climatic conditions. Warmer colors represent areas of higher suitability. Black circles represent points used for model training, and the red line delimits the current geographic distribution of the species. Map projected in Mollweide, datum WGS84

Modern climate Maxent models for *S. gracilis* indicated high suitability throughout the known distribution of this species with the exception of the “semidesert grassland” and “Chihuahua desert scrub” habitats (Gori & Enquist, 2003) of northern Mexico and New Mexico (Figure 3). However, these regions contain few records in our modeling occurrence dataset (Figure 3; Appendix S3, Table S3.1). Habitat suitability across the Intermountain West appears patchy and low despite a large number of records of *S. gracilis* from states such as Colorado and New Mexico. Here, models predict reduced suitability among the coniferous forests of the Rocky Mountains and big sagebrush steppe and shrubland of the Wyoming basin. The southern extent of suitable conditions for *S. gracilis* is characterized by a conspicuous break along the Trans‐volcanic Mexican Belt, lack of suitable conditions in the Yucatán Peninsula, and unsuitable conditions along the Pacific Coast of Central America (see Appendix S3, Fig. S3.2), regions with known occurrences of *Spilogale* typically attributed to the karyotypically distinct species *S. angustifrons* (Dragoo, 2009; Owen, Baker, & Williams, 1996).

#### 3.4.2 Historic models

Paleoecological niche models of *S. gracilis* show suitable conditions contracting southwards during times of maximal glacial extent and then expanding to mimic current distributions during interglacial periods (Figure 3). Clear separation of suitable conditions in the LGM appears in the southwestern USA and northwestern Mexico with two suitable patches split by what currently represents extents of both the Sonoran and Chihuahuan Deserts (Figure 3b). LGM models also predict areas of the Caribbean islands and Florida as maintaining suitable abiotic conditions during glacial maxima, although no records of any skunks exist for the Caribbean, and Florida is inhabited by the congener *S. putorius* (see Appendix S3, Fig. S3.2). In contrast to the LGM, LIG models predict increased areas of suitability across western USA and central USA/northern Mexico, although the patch of suitable conditions along the northwestern coast of Mexico, in Sonora, appears to be somewhat disconnected from those in the western USA and northern Mexico/southern Texas (Figure 3a).

## 4 Discussion

### 4.1 Pre‐Pleistocene divergence

Divergence estimates for *Spilogale* indicated that the TMRCA occurred between late Miocene and early Pliocene (*c*. 6.53 Ma with a 95% HPD of 8.64–4.34 Ma), a date that is concordant with estimates based on both molecular data (Eizirik et al., 2010) and the fossil record (Wang et al., 2014). Similar divergence was observed among antelope squirrels *Ammospermophilus* across the Miocene‐Pliocene boundary, where their three major lineages diverged across the warm deserts of North America (Mantooth et al., 2013). Following initial divergence in *Spilogale*, species‐level divergence between *S. putorius* and *S. gracilis* occurred during the late Pliocene to early Pleistocene (TMRCA *c*. 2.71 Ma with a 95% HPD of 4.06–1.70 Ma), a time period that corresponds to initial diversification in *Sorex* in northwestern North America (Hope, Panter, Cook, Talbot, & Nagorsen, 2014), diversification of *Xerospermophilus* from the Mojave Desert (Bell, Hafner, Leitner, & Matocq, 2010), and diversification of *Thomomys* across parts of the Great Basin (Belfiore, Liu, & Moritz, 2008). Thus, although PPD appears to have played a role in early stages of evolution among *Spilogale*, its role in shaping genetic diversity within species appears minimal, at least for *S. gracilis*.

Despite the lack of a strong signature of PPD on the genetic structure of *S. gracilis* as a whole, the genetic isolation of the Arizona clade does appear to partially correspond with well‐known barriers that pre‐date the Pleistocene (Figure 1, Numbers 1–4). Specifically, it appears that *S. gracilis* individuals within the Arizona clade were isolated from the West clade by the Bouse Embayment, a Late Miocene to Late Pliocene marine inundation along what is now the lower Colorado River (Hafner & Riddle, 2011; Riddle & Hafner, 2006). Although much older than divergence estimates for the Arizona clade, the recession of the Bouse Embayment (Figure 1, Number 1) did not occur until the Mid‐Late Pleistocene (Wood et al., 2013), a time that corresponds to diversification of both the Arizona and West clades (ca. 200 Ka). The Arizona clade also appears to have been isolated along other Pre‐Pleistocene filter barriers implicated in shaping regional mammalian evolution (Hafner & Riddle, 2011; Mathis, Hafner, & Hafner, 2014; Riddle & Hafner, 2006), including the Sierra Barabampo‐Rio Fuerte (Figure 1, Number 4) in the south and the Rio Grande‐Rio Conchos Rivers (Figure 1, Number 3) in the southeast. Interestingly, it is only along the Cochise filter barrier (Figure 1, Number 2), an Oligocene‐Miocene eruption of the Sierra Madre Occidental subjected to major vegetative fluctuations during QCC, that we see the potential for admixture among the three clades, although the absence of individuals from northern Arizona and the Colorado Plateau limit our inferences to other portions of the species’ range. When combined with the less compelling evidence for isolation provided by the ENM models, it does appear that at least for the Arizona clade, that PPD might have played a supporting role in shaping the genetic structure that we observed in extant populations of *S. gracilis*.

Interestingly, and in contrast to results observed for the Arizona clade, the West clade failed to show signatures of genetic isolation associated with well‐characterized PPD events. For example, major mountain ranges with orogenesis in the Cenozoic including the modern Rocky Mountains, Sierra Nevada, and Cascade Ranges appeared to have little impact on genetic structure within *S. gracilis*. This pattern contrasts with results for the related striped skunk, *Mephitis mephitis*, which showed distinct phylogeographic subdivisions across the Sierra Nevada Mountains (Barton & Wisely, 2012). Other co‐distributed mammals displaying phylogeographic breaks with mountains of the west include mule deer (Latch et al., 2014) and red foxes (Aubry, Statham, Sacks, Perrine, & Wisely, 2009; Volkmann, Statham, Mooers, & Sacks, 2015).

### 4.2 Quaternary climate change

Although patterns associated with the Arizona clade appear to support the potential impacts of PPD on *S. gracilis* genetic structure, phylogeographic patterns at the intraspecific level appear to have resulted more from QCC than PPD according to data from divergence estimates, demographic patterns, and ENM. Divergence estimates within *S. gracilis* indicate that East‐Central, Arizona, and West mtDNA lineages shared a common ancestor *c*. 1.36 Ma (95% HPD 2.11–0.822 Ma). This estimate, including the entire HPD, falls exclusively within the Pleistocene, supporting the conclusion that QCC associated with glacial expansion and contraction played a major role in shaping demographic histories and distributions of *S. gracilis* populations across western North America. Molecular dates and phylogenies, in conjunction with ENM of the LIG (~0.12 Ma), support the hypothesis that *S. gracilis* was isolated into three distinct refugia during interglacial periods.

Ecological niche models for the LIG highlighted three regions of high suitability for *S. gracilis*, Pacific Northwest coastline/central California, central Sonora Mexico, and northeastern Mexico/southern Texas (Figure 3a). Although suitable conditions appear contiguous between western USA and Sonora Mexico at the time of divergence, patchy areas of unsuitability exist west of the Colorado River and in central Arizona, potentially isolating these regions from one another. Similar to patterns obtained for present‐day climates, LIG models indicated low suitability across the grasslands of northwestern Mexico and southern New Mexico. Although ENMs provide corroborative evidence for effects of QCC on genetic structure in *S. gracilis*, one major advantage of using this method is to provide explicit hypotheses about the locations of putative glacial refugia during glacial maxima.

Our ENM for the LGM supported the presence of two and possibly three refugia for *S. gracilis* during the Quaternary (Figure 3b). The western USA, including much of California, coastal Oregon, and northern Baja California, appeared separated from a second area of high suitability in southern Texas and northeastern Mexico (i.e., Tamaulipas). These two regions would have supported two isolated populations of *S. gracilis* with limited gene flow during glacial maxima, leading to East‐Central and West genetic clades, respectively. A similar pattern, although with much less suitable areas along the west coast, was recently recovered for Bell's Vireo, *Vireo belleii* (Klicka, Kus, Title, & Burns, 2016), providing additional support for similar refugial patterns in other codistributed taxa. Using ENM reprojections to the LGM, Klicka et al. (2016) found support for two or potentially three Pleistocene refugia (i.e., central California, northwest Mexico, and northeast Mexico) that corresponded with phylogeographic breaks in mtDNA clades observed in *S. gracilis*.

While the distribution of suitable conditions during the LIG appears to support the presence of a potential isolated region in central Sonora (Arizona clade), LGM models did not yield such a distinctive pattern. The location of the third putative refugium isolating the Arizona clade from the West and East‐Central clades potentially occurred in one of two different areas (Figure 3b). The first potential Arizona clade refugium was located in central Arizona and southern Nevada separated from the California/West clade refugium by unsuitable conditions associated with Death Valley and the Mojave Desert. The second potential Arizona refugium was characterized by a north–south strip of suitable conditions located on the western side of the Sierra Madre Occidental but east of the Sinaloan Dry Forest (Rzedowski, 1978). Thus, LGM models did not clearly identify a single isolated patch of suitable conditions for the Arizona clade as they did for the West and East‐Central clades, but instead highlighted two potentially isolated patches of suitable conditions which could have potentially served as refugia for the Arizona clade. Although discrepancies as to the existence of a climatic refugium for the Arizona clades exist, ENM results support isolation of this population as early as the LIG with increased isolation during glacial maxima. These patterns are consistent with divergence time estimates and indicate that QCC, including both interglacial and glacial periods, functioned to shape the modern genetic diversity in extant populations of *S. gracilis* across western North America.

### 4.3 Signatures of secondary contact and dispersal across the desert southwest

One of the most important barriers separating fauna of the Chihuahuan and Sonoran Deserts is the Cochise filter barrier, a 1,500 m high plain spanning approximately 200 km (east–west) characterized by cool, transitional desert grasslands, known as the Deming Plains (Morafka, 1977). This barrier resulted in separation of desert lowland fauna throughout the Pleistocene, although oscillating climate cycles often led to expansion and contraction of grassland habitat which could explain why some species and not others show phylogeographic signatures associated with this barrier (Morafka, 1977). Phylogeographic breaks along this barrier have been recovered for bats (Weyandt & Van Den Bussche, 2007), birds (Klicka et al., 2016; Zink, Kessen, Line, & Blackwell‐Rago, 2001), small mammals, plants, and reptiles (Hafner & Riddle, 2011; Riddle & Hafner, 2006). Mitochondrial DNA indicates that the Cochise filter barrier could have helped to separate Arizona and East‐Central populations of *S. gracilis* throughout the Pleistocene (Figure 1). However, the occurrence of individuals belonging to the East‐Central clade in Arizona (west of the Rio Grande River and Deming Plains) as well as individuals belonging to the West clade in central New Mexico (east of the Rio Grande River) indicate secondary contact between these two mitochondrial clades has occurred across the desert southwest (Figure 1). This secondary contact appears to have been across the Deming Plains, which may have historically acted as a habitat‐based impediment to dispersing *S. gracilis* from either the eastern or western side of the barrier. Additional sampling and application of nuclear markers to specimens collected from the Deming Plains area (e.g., the Florida Mountains of New Mexico or south of Puerto Palomas, Chihuahua, Mexico) might help shed greater insight into patterns of secondary contact/isolation across this important Pleistocene barrier. The fact that a cool, semi‐arid grassland could function as a barrier to *S. gracilis* is further supported by results of our present‐day ENM: Predicted distributions of suitable conditions for *S. gracilis* under current climate conditions showed reduced suitability across the grasslands of the Apache Highlands ecoregion of southeastern Arizona and southwestern New Mexico as well as the grasslands of northern Mexico (Figure 3).

### 4.4 Biogeography of small mammals in western North America

Recent studies on small mammals with similar distributions to *S. gracilis* revealed similar patterns of intraspecific variation in both time and space to those seen in *S. gracilis*. The pallid bat, *Antrozous pallidus*, diverged during the Pliocene, followed by subsequent isolation of three unique mtDNA clades by QCC (Weyandt & Van Den Bussche, 2007). Geographic distributions of those mtDNA clades, which differed by 7% in Cytb sequences, were similar to those documented for *S. gracilis* with the exception of *A. pallidus* Clade B which included individuals from AZ, CO, and Baja California. Distributions of mtDNA clades of *A. pallidus* also closely matched the current ranges of three species of *Ammospermophilus* (Mantooth et al., 2013). A more recent study of range‐wide variation in the Cytb gene for the western harvest mouse, *Reithrodontomys megalotis*, found three distinct mtDNA clades estimated to have diverged during the early Pleistocene ~1.20 Ma (Nava‐García et al., 2016). Although lacking samples from AZ and NM, the East–West split seen in *R. megalotis* mirrored the split seen between our East‐Central and West clade in *S. gracilis*, providing support for at least two refugia during glacial maxima for small, western North American mammals.

### 4.5 Biogeography of small Carnivores in western North America

In comparison with similar, broadly distributed and generalist species of small carnivores, including other mephitid species (Barton & Wisely, 2012), *S. gracilis* displays a pattern more consistent with small mammals like rodents than similar or even smaller‐sized carnivores (Aubry et al., 2009; Dawson, Hope, Talbot, & Cook, 2014; Harding & Dragoo, 2012). For example, studies of striped skunks, *M. mephitis* (Barton & Wisely, 2012), ermine, *Mustela erminea* (Dawson et al., 2014), and red foxes, *Vulpes vulpes* (Aubry et al., 2009; Volkmann et al., 2015) all indicate recent divergences (<400 Ka) of intraspecific lineages following glaciation patterns across North America, but see Harding and Dragoo (2012). However, intraclade divergence within a western North America clade of the long‐tailed weasel, *Mustela frenata* appears more in line with patterns seen in *S. gracilis*, with major lineage divergence occurring around 1 Ma (Harding & Dragoo, 2012). Although none of these small carnivore studies used ENM to identify putative Pleistocene refugia, inferred refugial locations also differed from those identified for *S. gracilis*. However, striped skunks appear to have shared a similar refugium to *S. gracilis* individuals in the East‐Central clade in southern Texas and northeastern Mexico (Barton & Wisely, 2012). Comparisons with these studies support the hypothesis that *S. gracilis* responded to QCC differently than other, similar sized and related small carnivores, indicating a unique evolutionary history for this species across western North America.

## 5 Conclusions

Major phylogeographic patterns within *S. gracilis* appear to have resulted more from QCC than PPD. This pattern differs from other codistributed small carnivores, highlighting that individual species may respond differently to PPD and QCC, even when closely related (e.g., striped and spotted skunks). Our multifaceted approach highlights the complex response of *S. gracilis* populations to changing climates across the Quaternary, indicating that it was not glacial maxima alone that shaped genetic structure in this species, but instead a unique combination of both physiographic features (e.g., major rivers but not major mountain ranges) and temporally variable changes in habitat suitability associated with changing climates that best explain the observed variation. These patterns would have remained obscured in the absence of intense sampling across the three major regions characterizing western North America, highlighting the need for in depth sampling for species inhabiting distinct areas characterized by different biogeographic histories. Our results highlight the presence of additional Pleistocene refugia for small mammals (including small carnivores) in northwestern Mexico and provide additional support for intraspecific divergence among some western North American small mammals, including *S. gracilis*, around 1.0–1.2 Ma.

## Conflict of Interest

None declared.

## Author Contributions

AWF, RCD, LKA, GIG, and JWD conceived the ideas; AWF, GIG, JWD, and MR collected the data; AWF analyzed the niche modeling data; AWF and MMM analyzed the molecular data; MMM performed dating analysis; AWF led the writing with contributions from all coauthors.
