# Supplementary material for: Phylogeography of a widespread small carnivore, the western spotted skunk (Spilogale gracilis) reveals temporally variable signatures of isolation across western North America
Source: Ecol Evol. 2017 May 3;7(12):4229–40. doi: 10.1002/ece3.2931 (PMC5478080; doi:10.1002/ece3.2931)

**SUPPORTING INFORMATION**

Phylogeography of a widespread small carnivore, the western spotted skunk (*Spilogale gracilis*) reveals temporally variable signatures of isolation across western North America

Adam W. Ferguson, Molly M. McDonough, Gema I. Guerra, Margaret Rheude, Jerry W. Dragoo, Loren K. Ammerman, and Robert C. Dowler

**APPENDIX S1. Details on samples of the western spotted skunk, *Spilogale gracilis* used for generating DNA sequence data.**

**Table S1.1** Localities were *Spilogale gracilis* were sampled for this study including the collections responsible for loaning tissue samples and tissue identifiers (Tissue ID). The total number of base pairs for each of the three mtDNA genes are indicated together with major clade assignments. Collection abbreviations are as follows: ASNHC (Angelo State Natural History Collection), CMC (Centro de Investigación en Biodiversidad y Conservación Universidad Autónoma del Estado de Morelos), CIIDIR (Centro Interdisciplinario de Investigación para el Desarrollo Integral Regional, Unidad Durango, IPN, Durango), CIBM (Centro de Investigaciones Biológicas del Noroeste, S.C., La Paz, Baja California Sur), DMNS (Denver Museum of Nature and Science), ), IECR (Integral Ecology Research Center), JWD (Jerry W. Dragoo Institute for the Betterment of Skunks), MSB (Museum of Southwestern Biology, University of New Mexico), MWFB (Museum of Wildlife and Fish Biology, University of California, Davis), MZFC (Museo de Zoología "Alfonso L. Herrera" de la Facultad de Ciencias de la Universidad Nacional Autónoma de México), NMSU (New Mexico State University), TTU (Texas Tech University), UA (University of Arizona), UCD (University of California- Davis), UMNH (Utah Museum of Natural History, University of Utah). U = unknown

| **Tissue ID** | **Collection** | | **Collector** | **Country** | **State** | **Latitude (N)** | **Longitude (W)** | **Unc.**  **(m)*** | **Cytb** | **ND5** | **Dloop** | **Clade** |
| --- | --- | --- | --- | --- | --- | --- | --- | --- | --- | --- | --- | --- |
| *Spilogale gracilis* | | | | | | | | | | | | |
| CHC497 | UA | | M Rheude | USA | AZ | 32.014338 | 109.336884 | 5131 | 1140 | 630 | 420 | Arizona |
| HD73 | UA | | M Rheude | USA | AZ | 31.852105 | 111.002937 | 4564 | 1140 | 630 | 455 | Arizona |
| ASK5307 | ASNHC | | RC Dowler | USA | AZ | 31.775999 | 110.847832 | 92 | 1140 | - | 539 | Arizona |
| ASK6259 | ASNHC | | CE Ebeling | USA | AZ | 31.837688 | 110.905834 | 4691 | 1140 | - | 539 | Arizona |
| CAC681 | UA | | M Rheude | USA | AZ | 32.434661 | 110.906917 | 4586 | 1140 | - | 539 | Arizona |
| CAJD009 | UA | | M Rheude | USA | AZ | 32.451342 | 110.764005 | 19155 | 1140 | - | 533 | Arizona |
| CAL649 | UA | | M Rheude | USA | AZ | 32.371321 | 110.699946 | 7279 | 1140 | - | 539 | Arizona |
| CAS686 | UA | | M Rheude | USA | AZ | 32.316304 | 110.794262 | 59 | 1140 | - | 539 | Arizona |
| CHC468 | UA | | M Rheude | USA | AZ | 32.020310 | 109.349646 | 2377 | 1140 | - | 539 | Arizona |
| HD44 | UA | | M Rheude | USA | AZ | U | U | U | 1140 | - | 537 | Arizona |
| HD49 | UA | | M Rheude | USA | AZ | U | U | U | 1140 | - | 537 | Arizona |
| HD62 | UA | | M Rheude | USA | AZ | 32.158443 | 110.888446 | 121 | 1140 | - | 537 | Arizona |
| HD68 | UA | | M. Rheude | USA | AZ | 31.438279 | 110.097970 | 3204 | 1140 | - | 537 | Arizona |
| HD69 | UA | | M Rheude | USA | AZ | 31.886076 | 110.208869 | 4846 | 1140 | - | 537 | Arizona |
| HDSV20 | UA | | M Rheude | USA | AZ | 31.542327 | 110.294086 | 3636 | 1140 | - | 537 | Arizona |
| HDTuc27 | UA | | M Rheude | USA | AZ | 32.315925 | 110.973751 | 59 | 1140 | - | 537 | Arizona |
| HDTuc36 | UA | | M Rheude | USA | AZ | 32.137677 | 110.893704 | 2542 | 1140 | - | 537 | Arizona |
| NK103354 | MSB | | CC Hass | USA | AZ | 31.552800 | 110.347340 | 3737 | 1140 | - | 537 | Arizona |
| NK182242 | MSB | | WA Talbot | USA | AZ | 31.543445 | 110.748334 | 2912 | 1140 | - | 301 | Arizona |
| SRE531 | UA | | M Rheude | USA | AZ | 31.674162 | 110.950300 | 3737 | 1140 | - | 540 | Arizona |
| SRE598 | UA | | M Rheude | USA | AZ | 31.723002 | 110.970087 | 4638 | 1140 | - | 540 | Arizona |
| SRE599 | UA | | M Rheude | USA | AZ | 31.723002 | 110.970087 | 4638 | 1140 | - | 540 | Arizona |
| SRE600 | UA | | M Rheude | USA | AZ | 31.723002 | 110.970087 | 4638 | 1140 | - | 540 | Arizona |
| SRE690 | UA | | M Rheude | USA | AZ | 31.723002 | 110.970087 | 4638 | 1140 | - | 540 | Arizona |
| NK17574 | MSB | | JL Conwell | MEX | CHI | 30.833333 | 108.500000 | 3036 | 1140 | - | 537 | Arizona |
| NK42738 | MSB | | U | MEX | SON | 29.086790 | 112.071232 | 3400 | 1140 | - | 540 | Arizona |
| NK42868 | MSB | | U | MEX | SON | 29.086790 | 112.071232 | 3400 | 1140 | - | 540 | Arizona |
| CHP588 | UA | | M Rheude | USA | AZ | 31.884100 | 109.206254 | 3036 | 1140 | - | 537 | East-Central |
| CHR454 | UA | | M Rheude | USA | AZ | 31.757743 | 109.351579 | 4832 | 1140 | - | 537 | East-Central |
| NMW039 | MZFC | | LL Panguia | MEX | CHI | 28.215611 | 104.571611 | 3036 | 1140 | 618 | 540 | East-Central |
| NWM004 | MZFC | | LL Panguia | MEX | CHI | 28.215611 | 104.571611 | U | 1140 | 618 | 540 | East-Central |
| CRD8211 | CIIDIR | | CL Gonzalez | MEX | DGO | 23.465167 | 104.367000 | U | 1140 | - | 537 | East-Central |
| 06_872 | JWD | | JW Dragoo | USA | NM | 32.312220 | 106.777780 | 17307 | 1140 | - | 539 | East-Central |
| NK159567 | MSB | | P Polechla | USA | NM | 35.003936 | 106.644075 | 80 | 1140 | - | 537 | East-Central |
| NK3997 | MSB | | P Polechla | USA | NM | 33.795900 | 105.548917 | 1662 | 1140 | - | 540 | East-Central |
| TK22414 | TTU | | JR Holt | USA | NM | 33.670622 | 105.930779 | 1051 | 1140 | - | 497 | East-Central |
| 04_850 | JWD | | JW Dragoo | USA | NM | 32.312220 | 106.777780 | 17307 | 1140 | - | - | East-Central |
| 06_873 | JWD | | JW Dragoo | USA | NM | 32.312220 | 106.777780 | 17307 | 1140 | - | - | East-Central |
| 06_874 | JWD | JW Dragoo | | USA | NM | 32.312220 | 106.777780 | 17307 | 1140 | - | - | East-Central |
| 07_858 | JWD | | JW Dragoo | USA | NM | 32.312220 | 106.777780 | 17307 | 1140 | - | - | East-Central |
| ASK7849 | ASNHC | | Unknown | USA | TX | 29.370240 | 100.882297 | 50 | 1140 | 630 | 539 | East-Central |
| ASK7914 | ASNHC | | M Morris | USA | TX | 30.179030 | 101.019780 | 3400 | 1140 | 630 | 539 | East-Central |
| ASK8066 | ASNHC | | RC Dowler | USA | TX | 31.419500 | 100.450170 | 60 | 1140 | 618 | 429 | East-Central |
| ASK8069 | ASNHC | | M Morris | USA | TX | 30.440556 | 101.185278 | 2092 | 1140 | 618 | 537 | East-Central |
| ASK8369 | ASNHC | | AW Ferguson | USA | TX | 32.208840 | 101.475143 | 121 | 1140 | 618 | 537 | East-Central |
| TK119378 | TTU | | RD Bradley | USA | TX | 30.471705 | 99.781289 | 1624 | 1140 | 618 | 540 | East-Central |
| TK78766 | TTU | | RD Bradley | USA | TX | 29.613934 | 103.065700 | 3370 | 1140 | 618 | 539 | East-Central |
| TK79216 | TTU | | RD Bradley | USA | TX | 30.057594 | 103.505863 | 4585 | 1140 | 568 | 511 | East-Central |
| TK83167 | TTU | | RD Bradley | USA | TX | 30.549645 | 104.660679 | 4827 | 1140 | 618 | 539 | East-Central |
| ASK3709 | ASNHC | | J Wright | USA | TX | 31.438652 | 100.631756 | 3204 | 1140 | - | 539 | East-Central |
| ASK3990 | ASNHC | | P Maddox | USA | TX | 31.752519 | 100.286817 | 3055 | 1140 | - | 539 | East-Central |
| ASK4065 | ASNHC | | RC Dowler | USA | TX | 31.474093 | 100.538449 | 3636 | 1140 | - | 539 | East-Central |
| ASK4855 | ASNHC | | JG Brant | USA | TX | 29.931667 | 100.974999 | 66 | 1140 | - | 539 | East-Central |
| ASK5719 | ASNHC | | RC Dowler | USA | TX | 31.304892 | 101.105738 | 3036 | 1140 | - | 539 | East-Central |
| ASK6873 | ASNHC | | CE Ebeling | USA | TX | 29.903469 | 103.646969 | 3610 | 1140 | - | 539 | East-Central |
| ASK7157 | ASNHC | | CE Ebeling | USA | TX | 31.468889 | 100.535004 | 3636 | 1140 | - | 539 | East-Central |
| ASK7910 | ASNHC | | JP Karges | USA | TX | 30.665872 | 104.035444 | U | 1140 | - | 517 | East-Central |
| ASK9965 | ASNHC | | RC Dowler | USA | TX | 29.930724 | 100.943265 | 920 | 1140 | - | 537 | East-Central |
| ASK9985 | ASNHC | | B Martin | USA | TX | 29.264944 | 103.792320 | 2830 | 1140 | - | 537 | East-Central |
| JWD570 | JWD | | JW Dragoo | USA | TX | 31.528977 | 100.543188 | 3639 | 1140 | - | 513 | East-Central |
| ASK3059 | ASNHC | | T Carter | USA | TX | 31.305054 | 101.020797 | U | 705 | - | - | East-Central |
| ASK4853 | ASNHC | | J Campbell | USA | TX | 31.498333 | 100.090014 | 3639 | 705 | - | - | East-Central |
| ASK8487 | ASNHC | | R Brent | USA | TX | 29.757610 | 99.522140 | 2280 | 1140 | 429 | - | East-Central |
| TK83081 | TTU | | RD Bradley | USA | TX | 30.058000 | 103.506000 | 3400 | 1140 | 618 | - | East-Central |
| 1644 | CIBM | | Unknown | MEX | Baja | 23.480893 | 109.714399 | 2000 | 1140 | - | 539 | West |
| 1645 | CIBM | | Unknown | MEX | Baja | 24.143172 | 110.433256 | 2000 | 1140 | - | 539 | West |
| 1646 | CIBM | | Unknown | MEX | Baja | 23.480893 | 109.714399 | 2000 | 1140 | - | 539 | West |
| 5059 | CIBM | | Unknown | MEX | Baja | 23.480893 | 109.714399 | 2000 | 1140 | - | 539 | West |
| 4 | IECR | | M Gabriel | USA | CA | 41.147173 | 123.682000 | 5000 | 1140 | - | 457 | West |
| 5 | IECR | | M Gabriel | USA | CA | 40.003165 | 121.593022 | 5000 | 1140 | - | 455 | West |
| 6 | IECR | | M Gabriel | USA | CA | 40.003165 | 121.593022 | 5000 | 1140 | - | 449 | West |
| 15 | IECR | | M Gabriel | USA | CA | 41.147173 | 123.682000 | 5000 | 1140 | - | 456 | West |
| 22 | IECR | | M Gabriel | USA | CA | 40.003165 | 121.593022 | 5000 | 1140 | - | 457 | West |
| 24 | IECR | | M Gabriel | USA | CA | 40.003165 | 121.593022 | 5000 | 1140 | - | 457 | West |
| 27 | IECR | | M Gabriel | USA | CA | 40.003165 | 121.593022 | 5000 | 1140 | - | 457 | West |
| 41 | IECR | | M Gabriel | USA | CA | 41.147173 | 123.682000 | 5000 | 1140 | - | 457 | West |
| 42 | IECR | | M Gabriel | USA | CA | 41.147173 | 123.682000 | 5000 | 1140 | - | 457 | West |
| 48 | IECR | | M Gabriel | USA | CA | 37.028690 | 119.162654 | 5000 | 1140 | - | 457 | West |
| 49 | IECR | | M Gabriel | USA | CA | 37.028690 | 119.162654 | 5000 | 1140 | - | 457 | West |
| 61 | IECR | | M Gabriel | USA | CA | 37.028690 | 119.162654 | 5000 | 1140 | - | 457 | West |
| 79 | IECR | | M Gabriel | USA | CA | 41.147173 | 123.682000 | 5000 | 1140 | - | 454 | West |
| 86 | IECR | | M Gabriel | USA | CA | 41.147173 | 123.682000 | 5000 | 1140 | - | 444 | West |
| V20 | UCD | | DV Vuren | USA | CA | 38.184137 | 121.776755 | 576 | 1140 | - | 482 | West |
| V4 | UCD | | DV Vuren | USA | CA | 37.693739 | 119.728815 | 610 | 1140 | - | 456 | West |
| V6 | UCD | | DV Vuren | USA | CA | 34.019720 | 119.852780 | 657 | 1140 | - | 397 | West |
| 14 | IECR | | M Gabriel | USA | CA | 41.147173 | 123.682000 | 5000 | - | - | 456 | West |
| H2390 | JWD | | R Honeycutt | USA | CA | U^ǂ^ | U | U | 1140 | - | - | West |
| H2392 | JWD | | R Honeycutt | USA | CA | U | U | U | 1140 | - | - | West |
| V7 | UCD | | DV Vuren | USA | CA | 34.058933 | 119.920673 | 1000 | 705 | - | - | West |
| V8 | UCD | | DV Vuren | USA | CA | 33.996126 | 119.715677 | 174 | 705 | - | - | West |
| 13613 | DMNS | | E Whitehead | USA | CO | 37.208899 | 108.492030 | 6050 | 1140 | - | 494 | West |
| TK182721 | TTU | | RD Bradley | USA | NM | 33.677821 | 105.927262 | 30 | 1140 | - | 458 | West |
| WT179 | NMSU | | J Frey | USA | NM | 33.604871 | 107.151450 | 30 | 1140 | - | 502 | West |
| 31867 | UMNH | | EA Rickart | USA | NV | 40.164020 | 115.503970 | 300 | 1140 | - | 427 | West |
| 31868 | UMNH | | EA Rickart | USA | NV | 40.164020 | 115.503970 | 300 | 1140 | - | 458 | West |
| ASK6778 | ASNHC | | CE Ebeling | USA | OR | 42.159597 | 124.141773 | 1807 | 1140 | - | 539 | West |
| 13615 | DMNS | | J Leuders | USA | WY | 44.181741 | 106.945466 | 200 | 1140 | - | 494 | West |
| *Spilogale putorius* | | | | | | | | | | | | |
| ASK7814 | ASNHC | | F Collins | USA | TX | 29.893333 | 96.013056 | U | 1140 | 628 | 510 | Outgroup |
| *Spilogale pygmaea* | | | | | | | | | | | | |
| TK45070 | TTU | | U | MEX | MIC | 18.096343 | 102.754015 | U | 1140 | 643 | 539 | Outgroup |
| *Conepatus leuconotus* | | | | | | | | | | | | |
| ASK8091 | ASNHC | | AW Ferguson | MEX | MOR | 18.507090 | 102.754015 | 10 | 1140 | 648 | 539 | Outgroup |
| *Mephitis mephitis* | | | | | | | | | | | | |
| WFB8990 | WFB | | AJ Liddell | USA | TX | 30.528856 | 103.829531 | 30 | 1140 | 659 | 453 | Outgroup |
| *Mydaus javanensis* | | | | | | | | | | | | |
| AB564095.1 | Genbank | | JS Sato | IND | Java | U | U | U | 1140 | - | 536 | Outgroup |

^ǂ^U = unknown

* = Coordinate Uncertainty in metres

**APPENDIX S2. Details regarding DNA sequencing protocols and primers used to generate mitochondrial sequence data from samples of western spotted skunk, *Spilogale gracilis.***

## Gene amplification

### Mitochondrial datasets

Mitochondrial cytochrome*-b* (1140 base pairs; Cytb) was amplified using universal primers LGL765 and LGL766 (Bickham *et al.*, 1995), NADH dehydrogenase 5 (629 bps; ND5) and control region (503 bps; D-loop) using carnivore-specific primers ND5-DF1 and ND5-DR1 (Trigo *et al*., 2008), and L16272 and H1008 (Arnason & Johnsson, 1992), respectively. Additional primers developed for *Conepatus leuconotus* were used to amplify smaller fragments of the D-loop gene to avoid the complicated repeat region characterizing the control region. These primer pairs included L398 and H601 and L724 and H1008 (Dragoo *et al.*, 2003). Details for primer sequences are provided below (Table S2.1).

The total reaction volume of 25 µl included 50-100 ng of DNA, 0.12 µM of each primer, 1.5 mM MgCl2, 0.012 mM each dNTPs, 1X reaction buffer, 0.32 mg/mL Bovine Serum Albumin, and 0.625 U GoTaq *Taq* polymerase (Promega Corporation, Madison, Wisconsin, USA). Thermal profiles for amplification of tissue samples were as follows. For Cytb the thermal profile included an initial denature at 95^o^C for 3 min followed by 34 cycles of 95^o^C for 1 min, 50^o^C for 1 min, and 72^o^C for 1 min, followed by a final extension time of 72^o^C for 10 min. The thermal profile for ND5 began with a 10 cycle touchdown, each of which had a 30 s denaturing step at 94 °C, 30 s annealing at 60–51 °C, and 1min extension at 72 °C, followed by 30–34 cycles of 30 s denaturing at 94 °C, 30 s annealing at 50 °C and 1min extension at 72 °C. The thermal profile for D-Loop included an initial denature at 95^o^C for 3 min followed by 34 cycles of 95^o^C for 1 min, 48^o^C for 1 min, and 72^o^C for 1 min, followed by a final extension time of 72^o^C for 10 min. All PCR reactions included negative controls using water in lieu of DNA samples. PCR products were electrophoresed on 1% agarose gels containing ethidium bromide and viewed under ultraviolet light to verify successful amplification. Ethanol precipitation was used to clean up PCR products.

## Cycle sequencing

Cycle sequencing was performed with BigDye version 3.1. (Applied Biosystems, Inc., Foster City, California, USA) and reaction conditions followed manufacturers protocols. Sequences were electrophoresed on and ABI 3130 (Applied Biosystems, Inc., Foster City, California, USA) sequencing platform.

**Table S2.1.** Primers used PCR and sequencing of mitochondrial genes in the western spotted skunk, *Spilogale gracilis*.

| **Primer Name** | **Primer sequence** | **T_m_** |
| --- | --- | --- |
| Cytb | |  |
| LGL765 | 5'-GAAAAACCATCGTTGTWATTCAACT-3' | 52.6°C |
| LGL766 | 5'-GTTTAATTAGAATYTYAGCTTTGGG-3' | 50.7°C |
|  |  |  |
| ND5 | |  |
| ND5-DF1 | 5'-TTGGTGCAACTCCAAATAAAAGT-3' | 53.4°C |
| ND5-DR1 | 5'-AGGAGTTGGGCCTTCTATGG-3' | 56.5°C |
|  |  |  |
| D-Loop | |  |
| L16272 | 5'-TACACTGGTCTTGTAAAAC-3' | 49.1°C |
| L724 | 5'-CCGCCAAACCCCAAAAA-3' | 54.0°C |
| H1008 | 5'-AAGGCTAGGACCAAACCT-3' | 53.1°C |
| L398 | 5'-CCTCTCAAATGGGACATCTCG-3' | 55.1°C |
| H601 | 5'-GGTTGTATGATGCGGGTAAATG-3' | 54.1°C |

##

## References

Arnason, U., & Johnsson, E. (1992) The complete mitochondrial DNA sequence of the harbor seal, *Phoca vitulina*. *Journal of Molecular Evolution* **34**, 493–505.

Bickham, J.W., Wood, C.C., & Patton, J.C. (1995) Variation in mitochondrial cytochrome b sequences and allozymes in sockeye (*Oncorhynchus* *nerka*). *Journal of Heredity* **86**, 140–144.

Dragoo, J.W., Honeycutt, R.L., & Schmidly, D.J. (2003) Taxonomic status of white-backed hog-nosed skunks, genus *Conepatus* (Carnivora : Mephitidae). *Journal of Mammalogy* **84**, 159–176.

Trigo, T.C., Freitas, T.R.O., Kunzler, G., Cardoso, L., Silva, J.C.R., Johnson, W.E., O’Brien, S.J., Bonatto, S.L., & Eizirik, E. (2008) Inter-species hybridization among Neotropical cats of the genus *Leopardus*, and evidence for an introgressive hybrid zone between *L*. *geoffroyi* and *L*. *tigrinus* in southern Brazil. *Molecular Ecology* **17**, 4317–4333.

**APPENDIX S3. Details on museum records (Table S3.1) used for generating ecological niche modeling as well as figures depicting the area used for model training (‘M’, Fig. S3.1) and ecological niche models projected across North America (Fig. S3.2).**

**Table S3.1** Specimen data for 84 records of *Spilogale gracilis* used to generate ecological niche models. Collection abbreviations are as follows: ASNHC (Angelo State Natural History Collection), CIBM (Centro de Investigaciones Biológicas del Noroeste, S.C.), CRD (Instituto

Politecnico Nacional, Colección Cientifica de Fauna Silvestre), DMNS (Denver Museum of Nature and Science), KU (University of Kansas), LACM (Los Angeles County Museum), MSB (Museum of Southwestern Biology), MVZ (Museum of Vertebrate Zoology), MWFB (Museum of Wildlife and Fish Biology, UC Davis), MCFZ (Colección de Mamíferos del Museo de Zoología Alfonso L . Herrera), UMMZ (University of Michigan Museum of Zoology), UMNH (Utah Museum of Natural History), UNAM (Universidad Nacional Autónoma de México, Colección Nacional de Mamíferos), USNM (United States National Museum), and UWBM (University of Washington, Burke Museum).

| **Collection** | **Specimen Number** | **Species** | **Subspecies** | **Collection Date** | **Country: State; County/Mpio** | **Specific Locality** | **Latitude (N)** | **Longitude (W)** | **Coordinate Uncertainty (m)** | **Collector** |
| --- | --- | --- | --- | --- | --- | --- | --- | --- | --- | --- |
| LACM | LACM  31162 | *Spilogale gracilis* | *amphialus* | 5 Dec 1941 | USA: CA; Santa Barbara | Santa Rosa Id; Corral Canyon | 33.939086 | 120.110924 | 1609 | Couffer, J. |
| LACM | LACM  30160 | *Spilogale gracilis* | *amphialus* | 99 XXX 9999 | USA: CA; Santa Barbara | Santa Cruz Id | 34.032420 | 119.770862 | 1609 | Luchetti |
| USNM | USNM  225134 | *Spilogale gracilis* | *gracilis* | 11 Feb 1916 | USA: CO | Shanwnee | 39.421110 | 105.553610 | 3036 | Gibbs, C. D. |
| USNM | USNM  151418 | *Spilogale gracilis* | *gracilis* | 4 Jan 1908 | USA: CO | Coventry | 38.159663 | 108.365029 | 3036 | Smith, C. H. |
| USNM | USNM  224033 | *Spilogale gracilis* | *gracilis* | 24 Jan 1914 | USA: CO | Bondad | 37.059530 | 107.873157 | 3036 | Patrick, W. E. |
| USNM | USNM  272037 | *Spilogale gracilis* | *gracilis* | 24 Jan 1942 | USA: ID;  Boise | Horseshoe Bend | 43.914720 | 116.196940 | 1584 | Marshal, W. H. |
| USNM | USNM  128945 | *Spilogale gracilis* | *gracilis* | 5 Sep 1903 | USA: NM | Raton Range, Folson, Oak Canon | 36.902871 | 103.898263 | 3813 | Howell, A. H. |
| USNM | USNM  221886 | *Spilogale gracilis* | *gracilis* | 2 Feb 1916 | USA: NV | Spaulding Canyon | 40.563060 | 117.779440 | 1506 | Guthrie, J. D. |
| USNM | USNM  221886 | *Spilogale gracilis* | *gracilis* | 2 Feb 1916 | USA: NV | Spaulding Canyon | 40.563060 | 117.779440 | 1506 | Guthrie, J. D. |
| USNM | USNM  222504 | *Spilogale gracilis* | *gracilis* | 23 Sep 1910 | USA: OR | Narrows | 43.279170 | 118.961110 | 3036 | Becker, R. H. |
| USNM | USNM  274331 | *Spilogale gracilis* | *gracilis* | 22 Jul 1939 | USA: OR; | Baker | 44.775000 | 117.833330 | 4037 | Scheffer, V. B. |
| USNM | USNM  78883 | *Spilogale gracilis* | *gracilis* | 29 Jun 1898 | USA: OR;  Lake | Plush | 42.411670 | 119.902780 | 3036 | Streator, C. P. |
| USNM | USNM  78884 | *Spilogale gracilis* | *gracilis* | 30 Jun 1896 | USA: OR;  Lake | Plush, Warner Lake | 42.411670 | 119.902780 | 3036 | Streator, C. P. |
| USNM | USNM  188463 | *Spilogale gracilis* | *gracilis* | 2 Jan 1889 | USA: UT | St. George | 37.097363 | 113.571853 | 3250 | Bailey, V. |
| USNM | USNM  160115 | *Spilogale gracilis* | *gracilis* | 1 Jul 1909 | USA: WY | Chugwater | 41.756670 | 104.821110 | 3062 | Cary, M. |
| USNM | USNM  171336 | *Spilogale gracilis* | *gracilis* | 30 May 1911 | USA: WY | Fort Steele | 41.778060 | 106.945830 | 3036 | Anthony, H. E. |
| USNM | USNM  98277 | *Spilogale gracilis* | *gracilis* | 28 Jul 1899 | MEX: CHI | Colonia Garcia, Near | 29.974991 | 108.337291 | 3036 | Nelson, Goldman, E. A. |
| USNM | USNM  157117 | *Spilogale gracilis* | *gracilis* | 29 Jul 1908 | USA: NM | Animas Mountains, N Slope Animas Peak | 31.582537 | 108.787930 | 4691 | Goldman, E. A. |
| DMNS | DMNS  13615 | *Spilogale gracilis* | *gracilis* | 31 Dec 2000 | USA: WY; Johnson | Caribou Creek, UTM Zone 13T, 344500E, 4893899N | 44.181741 | 106.945466 | 200 | Leuders, J. |
| KU | KU  48054 | *Spilogale gracilis* | *gracilis* | 25 Dec 1930 | USA: NV;  Elko | Ruby Mts; 4 mi E of Lee; Kleckner Creek | 40.572220 | 115.542500 | 500 | Borell, A. E. |
| KU | KU  48057 | *Spilogale gracilis* | *gracilis* | Winter 1928 | USA: NV;  Elko | Ruby Mts; 4 mi E of Lee; Kleckner Creek | 40.572220 | 115.542500 | 500 | Borell, A. E. |
| MVZ | MVZ  78392 | *Spilogale gracilis* | *gracilis* | 25 May 1937 | USA: CA; Lassen | 4 mi WNW Stacy | 40.257320 | 120.101570 | 3219 | Henry S. Fitch, H. S. |
| MVZ | MVZ  47294 | *Spilogale gracilis* | *gracilis* | 5 Aug 1931 | USA: CA; Modoc | near Boles Spring Ranger Station, 30 mi N Alturas | 41.754629 | 120.791501 | 470 | Russell, W. C. & Jordan, A. A. |
| MVZ | MVZ  34277 | *Spilogale gracilis* | *gracilis* | 28 Oct 1924 | USA: CA; Placer | shore Lake Tahoe, 2 mi S mouth Truckee River | 39.139118 | 120.152498 | 4099 | Borell, A. E. & Mofitt, J. M. |
| MVZ | MVZ  51858 | *Spilogale gracilis* | *gracilis* | 29 Aug 1931 | USA: ID; Bannock | Justice Park | 42.692200 | 112.377800 | 14 | Whitlow, W. B. |
| MVZ | MVZ  69702 | *Spilogale gracilis* | *gracilis* | 21 Dec 1935 | USA: ID; Blaine | Wood River, 1 mi N Ketchum | 43.695300 | 114.362800 | 4339 | Twining, H. T. & Linderman, A. |
| MVZ | MVZ  67256 | *Spilogale gracilis* | *gracilis* | 17 Apr 1905 | USA: ID; Gooding | 2 mi S Hagerman | 42.783200 | 114.897800 | 4477 | Davis, W. B. |
| MVZ | MVZ  37304 | *Spilogale gracilis* | *gracilis* | 1 Jan 1927 | USA: NV;  Nye | Millett Post Office | 39.015280 | 117.180000 | 428 | Alexander, A. M. et al. |
| MVZ | MVZ  45902 | *Spilogale gracilis* | *gracilis* | 22 Aug 1938 | USA: NV; White Pine | 0.25 mi W Hamilton | 39.253060 | 115.490220 | 2174 | Hall, E. R. |
| UMNH | UMNH  8802 | *Spilogale gracilis* | *gracilis* | 16 Feb 1953 | USA: UT; Duschene | 4 mi. NW Duschene | 40.204400 | 110.454800 | 3817 | Nelsen, W. |
| UMNH | UMNH  13762 | *Spilogale gracilis* | *gracilis* | 19 Aug 1956 | USA: UT; Emery | Upper Joe's Valley | 39.415000 | 111.244000 | 2012 | Rasmussen, D. I. |
| UMNH | UMNH  7616 | *Spilogale gracilis* | *gracilis* | 25 Aug 1950 | USA: UT;  Juab | Above Jct. Birch & Trout Creeks | 39.709620 | 113.864040 | 3232 | Hansen, R. M. |
| UWBM | UWBM  38625 | *Spilogale gracilis* | *gracilis* | 4 Aug 1991 | USA: OR; Harney | Malheur National Wildlife Refuge; Hwy 205 at the Narrows | 43.279200 | 118.961100 | 14 | B. D'Veck |
| USNM | USNM  207675 | *Spilogale gracilis* | *latifrons* | 13 Apr 1915 | USA: OR | Millers, Mouth Of Deschutes River | 45.635560 | 120.913330 | 3201 | Jewett, S. G. |
| USNM | USNM  223802 | *Spilogale gracilis* | *latifrons* | 17 Sep 1916 | USA: OR | Hampton | 43.672780 | 120.232780 | 3036 | Snyder, W. C. |
| ASNHC | ASNHC  13005 | *Spilogale gracilis* | *latifrons* | 24 Jun 2004 | USA: OR; Curry | Siskiyou National Forest near Big Redwood Camp, 10T 0405653, 4668083 | 42.159597 | 124.141773 | 500 | Ebeling, C. E. |
| KU | KU  143990 | *Spilogale gracilis* | *latifrons* | 16 Mar 1990 | USA: OR; Lincoln | Nortons, 3 mi N, 3 mi W of: T10S R9W SEC13 | 44.700000 | 123.730000 | 1152 | Hacker, A. |
| LACM | LACM  30163 | *Spilogale gracilis* | *latifrons* | 99 XXX 9999 | USA: CA | Edgewood | 41.456146 | 122.430297 | 1112 | Jewett |
| UMMZ | UMMZ  107954 | *Spilogale gracilis* | *latifrons* | 14 Mar 1938 | USA: WA; Clallam | Ozette, Coal Cr. | 47.976950 | 124.592410 | 4197 | Clev. Mus. Nat. Hist. |
| USNM | USNM  66990 | *Spilogale gracilis* | *latifrons* | 31 Jul 1894 | CAN: British Columbia | Port Moody | 49.283333 | 122.850000 | 5634 | Streator, C. P. |
| USNM | USNM  228528 | *Spilogale gracilis* | *latifrons* | 14 Jun 1916 | USA: OR | Oakridge, Hall Creek | 43.746670 | 122.460560 | 4098 | Clark, E. |
| USNM | USNM  87052 | *Spilogale gracilis* | *latifrons* | 10 Mar 1905 | USA: OR; | Marmot | 45.396670 | 122.114440 | 3036 | Aschoff, A. |
| USNM | USNM  233628 | *Spilogale gracilis* | *latifrons* | 29 Jan 1919 | USA: WA; Jefferson | Duckabush, Hood'S Canal | 47.652220 | 122.930830 | 4271 | Cantwell, G. G. |
| USNM | USNM  230110 | *Spilogale gracilis* | *latifrons* | 6 Sep 1918 | USA: WA; Snohomish | Index | 47.820830 | 121.553890 | 912 | Taylor, W. P. |
| UWBM | UWBM  20193 | *Spilogale gracilis* | *latifrons* | 18 Mar 1959 | USA: OR; Columbia | Keasey, 0.5 mi N, 0.5 mi E | 45.868900 | 123.318000 | 1152 | L. A. Fredrich |
| CRD | CRD  3000 | *Spilogale gracilis* | *leucoparia* | 13 Sep 2000 | MEX: DUR; Santiago Pap. | 0.4 km S, 0.35 km W San Juan de Camarones | 24.922660 | 106.417000 | 50 | Muniz, R. M. |
| USNM | USNM  79587 | *Spilogale gracilis* | *leucoparia* | 28 Jul 1896 | MEX: COA | Sierra Encarnacion | 24.783260 | 101.261935 | 9263 | Nelson, Goldman, E. A. |
| USNM | USNM  116946 | *Spilogale gracilis* | *leucoparia* | 6 May 1902 | MEX: COA; | Saltillo | 25.448563 | 100.958706 | 7324 | Nelson, Goldman, E. A. |
| USNM | USNM  214348 | *Spilogale gracilis* | *leucoparia* | 23 Jun 1916 | USA: AZ | Congress Junction | 34.157687 | 112.851025 | 3896 | Taylor, W. P. |
| ASNHC | ASNHC  13557 | *Spilogale gracilis* | *leucoparia* | 29 Jul 2008 | USA: TX; Howard | 2 mi S Big Spring on U.S. 87, N 32.20884, W 101.47524, 797 m elev. | 32.208840 | 101.475143 | 100 | Ferguson, A. W. |
| ASNHC | ASNHC  13558 | *Spilogale gracilis* | *leucoparia* | 19 Jul 2008 | USA: TX;  Jeff Davis | Hwy 118 near McDonald Observatory, Mt Locke; 13R 0592404, 3392970 | 30.665872 | 104.035444 | 100 | Karges, J. P. |
| CRD | CRD 8211 | *Spilogale gracilis* | *leucoparia* | 12 May 2006 | MEX: DUR; Mezquital | Costado del Balneario "La Joya" | 23.465167 | 104.367000 | 81 | Gonzalez, C. L. |
| KU | KU  45029 | *Spilogale gracilis* | *leucoparia* | 1 Mar 1952 | MEX: COA | Boquillas, 7 mi S, 2 mi E | 29.086040 | 102.903200 | 4585 | Baker, R. H. |
| KU | KU  82823 | *Spilogale gracilis* | *leucoparia* | 11 May 1960 | MEX: CHI | URIQUE | 27.212500 | 107.913890 | 920 | Anderson, S. |
| KU | KU  89151 | *Spilogale gracilis* | *leucoparia* | 23 Nov 1961 | MEX: NUL | Linares, 35 mi S of' Ibarrilla | 24.370000 | 99.670000 | 376 | Clifton, P. L. |
| KU | KU  48046 | *Spilogale gracilis* | *leucoparia* | 1 Nov 1929 | USA: AZ; Yuma | Tinajas Altas; Gila Mts | 32.311700 | 114.050800 | 14 | Bailey, B. |
| KU | KU  48046 | *Spilogale gracilis* | *leucoparia* | 1 Nov 1929 | USA: AZ; Yuma | Tinajas Altas; Gila Mts | 32.310000 | 114.050000 | 14 | Bailey, B. |
| LACM | LACM  7979 | *Spilogale gracilis* | *leucoparia* | 13 Feb 1942 | USA: CA; Riverside | Riverside Mts | 34.022829 | 114.544032 | 4828 | Stager, K. E. |
| MSB | MSB  160345 | *Spilogale gracilis* | *leucoparia* | 1 Feb 1953 | USA: AZ;  Gila | 3 mi S, 5 mi W Roosevelt | 33.624021 | 111.221041 | 1051 | Van Gelder, R. G. |
| MSB | MSB  160330 | *Spilogale gracilis* | *leucoparia* | 21 July 1954 | USA: AZ; Coconino | Bright Angel Trail, 1.2 mi below Indian Garden, Grand Canyon National Park | 36.094950 | 112.112520 | 1241 | Davis, W. H. |
| MSB | MSB  60677 | *Spilogale gracilis* | *leucoparia* | 9 Sep 1982 | USA: NM; Santa Fe | Sante Fe near Jct Colorado St and St Francis St, B | 35.686943 | 105.937233 | 1609 | Mackey, R. |
| MSB | MSB  214933 | *Spilogale gracilis* | *leucoparia* | 11 Aug 2009 | USA: AZ; Santa Cruz | Patagonia on Harshaw Creek Road | 31.543445 | 110.748334 | 1802 | Talbot, W. A. |
| MSB | MSB  214944 | *Spilogale gracilis* | *leucoparia* | 27 Sep 2005 | USA: NM; Bernalillo | Interstate 25 at Tijeras Arroyo, ca 0.75 mi. NE. Albuquerque City Limits | 35.002018 | 106.644283 | 10 | Polechla, P. J. & Hawkins, M. C. |
| MVZ | MVZ  10573 | *Spilogale gracilis* | *leucoparia* | 13 May 1910 | USA: CA; Imperial | Pilot Knob, Colorado River | 32.731400 | 114.749200 | 1000 | Stephens, F. |
| MVZ | MVZ  55214 | *Spilogale gracilis* | *leucoparia* | 21 Aug 1932 | USA: AZ; Navajo | Oraibi | 35.876400 | 110.639700 | 5003 | Alexander, A. M. |
| MVZ | MVZ  64980 | *Spilogale gracilis* | *leucoparia* | 18 Oct 1934 | USA: CA;  Inyo | 0.5 mi SW Junction Ranch, 16 mi SSE Darwin | 36.065444 | 117.515063 | 30 | Hill, J. E. |
| MVZ | MVZ  69959 | *Spilogale gracilis* | *leucoparia* | 6 Feb 1936 | USA: NV; Clark | 1.5 mi NW Boulder City | 35.993990 | 114.850600 | 5123 | Smiley, D. C. |
| MZFC | NMW  39 | *Spilogale gracilis* | *leucoparia* | Unknown | MEX: CHI; Comargo | Rancho Mestinas | 28.215611 | 104.571611 | 66 | Panguina, L. L. |
| UNAM | CNMA  3265 | *Spilogale gracilis* | *leucoparia* | 20 Dec 1957 | MEX: SON; Carbo | 23 mi SE Carbo, Cueva del Tigre | 29.446937 | 110.694883 | 14930 | Unknown |
| USNM | USNM  117208 | *Spilogale gracilis* | *leucoparia* | 6 Jul 1902 | USA: TX | Eagle Pass | 28.711600 | 100.485437 | 4087 | Nelson, Goldman, E. A. |
| USNM | USNM  126372 | *Spilogale gracilis* | *leucoparia* | 31 Mar 1903 | USA: TX | Langtry | 29.808330 | 101.558330 | 3036 | Gaut, J. H. |
| USNM | USNM  188467 | *Spilogale gracilis* | *leucoparia* | 13 Jan 1886 | USA: TX;  Mason | Mason | 30.748610 | 99.230280 | 3204 | Henry, I. B. |
| USNM | USNM  186452 | *Spilogale gracilis* | *leucoparia* | 2 Dec 1885 | USA: TX; Mason | Mason | 30.748610 | 99.230280 | 3204 | Henry, I. B. |
| USNM | USNM  79079 | *Spilogale gracilis* | *lucasana* | 7 Nov 1905 | MEX: Baja California Sur | Comondu | 26.058761 | 111.824165 | 3036 | Nelson, Goldman, E. A. |
| CIB | CIB  1645 | *Spilogale gracilis* | *lucasana* | 7 Nov 2002 | MEX: Baja California | La Paz; El Comitan | 24.143172 | 110.433256 | 3045 | Gonzalez-Quintero, P. |
| MVZ | MVZ  49502 | *Spilogale gracilis* | *lucasana* | 5 Apr 1931 | MEX: Baja CA Sur | Comondu | 26.062500 | 111.812500 | 1725 | Lamb, C. C. |
| USNM | USNM  139756 | *Spilogale gracilis* | *martirensis* | 12 Oct 1905 | MEX: Baja California Sur | San Ignacio | 28.739580 | 113.842966 | 3036 | Nelson, Goldman, E. A. |
| MVZ | MVZ  2340 | *Spilogale gracilis* | *martirensis* | 18 Sep 1908 | USA: CA; Orange | Trabuco Canyon, Santa Ana Mts. | 33.676075 | 117.516559 | 30 | Wilder, H. E. |
| MVZ | MVZ  18852 | *Spilogale gracilis* | *martirensis* | 4 July 1912 | USA: CA;  San Diego | La Puerta Valley | 32.970000 | 116.440000 | 1000 | Stephens, F. |
| MVZ | MVZ  24825 | *Spilogale gracilis* | *phenax* | 13 Sep 1916 | USA: CA; Fresno | Kings River Ca?on | 36.793800 | 118.581000 | 2416 | White, H. G. |
| MVZ | MVZ  44280 | *Spilogale gracilis* | *phenax* | 3 Apr 1930 | USA: CA;  Los Angeles | near Lovejoy Buttes | 34.602267 | 117.849936 | 4828 | Seth B. Benson, S. B. |
| MVZ | MVZ  25344 | *Spilogale gracilis* | *phenax* | 8 May 1917 | USA: CA; Mendocino | Laytonville | 39.686337 | 123.485194 | 1609 | Clarke, F. C. |
| MWFB | V20 | *Spilogale gracilis* | *phenax* | Unknown | USA: CA; Solano | HWY 12, 5 mi NW Rio Vista | 38.184137 | 121.776755 | 4753 | Van Vuren, D. |
| UMMZ | UMMZ  54721 | *Spilogale gracilis* | *phenax* | 25 Sep 1922 | USA: CA; Kings | Lemoore | 36.302195 | 119.781785 | 3714 | Dice, L. R. |

**Figure S3.1** Depiction of ‘M’ or the delimited area used to generate ecological niche model results (thick, black polygon). This polygon represents a bounded area which limited sampling of background points and environmental conditions to this region during model development in Maxent. The current range of *Spilogale gracilis* is identified by the thin, red polygon and a series of 752 georeferenced museum specimens of *S. gracilis* from the MaNIS database are depicted by the gray circles.


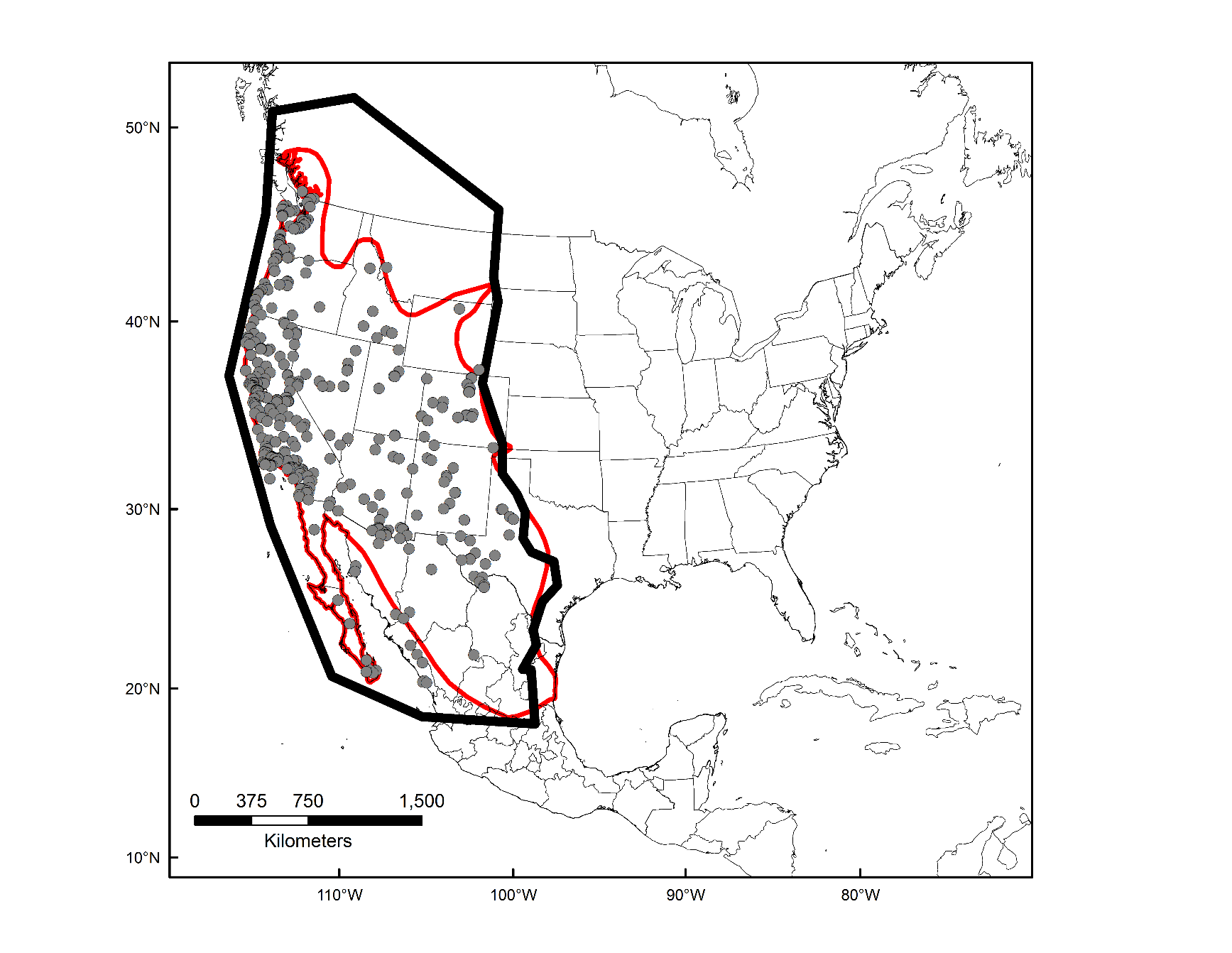


**Figure S3.2** Ecological niche models generated using 84 records of *Spilogale gracilis* and all 19 bioclimatic variables in the program Maxent projected across North America under last interglacial (a), last glacial maximum (b), and present day climatic conditions. The red polygon represents the current geographic range of *S. gracilis* and the black dots individual records of the 84 used to generate models (c).


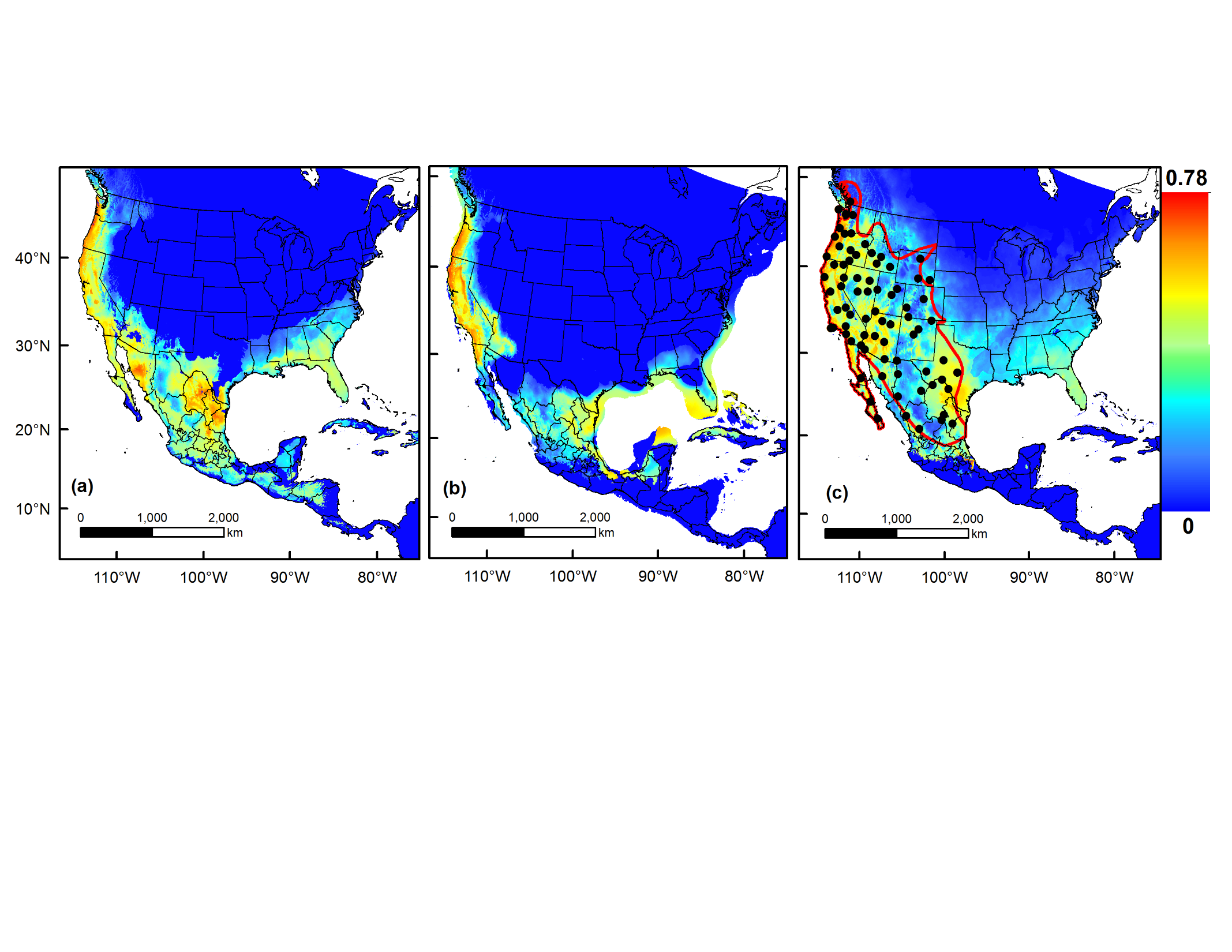

Supplement: Supplementary file 1 [file ECE3-7-4229-s001.docx]
